# Reduction of [^68^Ga]Ga-DOTA-TATE injected activity for digital PET/MR in comparison with analogue PET/CT

**DOI:** 10.1186/s40658-024-00629-z

**Published:** 2024-03-15

**Authors:** Christina P. W. Cox, Tessa Brabander, Erik Vegt, Quido G. de Lussanet de la Sablonière, Laura H. Graven, Frederik A. Verburg, Marcel Segbers

**Affiliations:** Department of Radiology and Nuclear Medicine, Erasmus MC, University Medical Center Rotterdam, Postbus 2040, 3000 CA Rotterdam, The Netherlands

**Keywords:** Gallium Ga 68 dotatate, Positron emission tomography computed tomography, Positron emission tomography, PET/MRI, BSREM, Block-sequential regularized expectation maximization, Image quality, Neuroendocrine tumour

## Abstract

**Background:**

New digital detectors and block-sequential regularized expectation maximization (BSREM) reconstruction algorithm improve positron emission tomography (PET)/magnetic resonance (MR) image quality. The impact on image quality may differ from analogue PET/computed tomography (CT) protocol. The aim of this study is to determine the potential reduction of injected [^68^Ga]Ga-DOTA-TATE activity for digital PET/MR with BSREM reconstruction while maintaining at least equal image quality compared to the current analogue PET/CT protocol.

**Methods:**

NEMA IQ phantom data and 25 patients scheduled for a diagnostic PET/MR were included. According to our current protocol, 1.5 MBq [^68^Ga]Ga-DOTA-TATE per kilogram (kg) was injected. After 60 min, scans were acquired with 3 (≤ 70 kg) or 4 (> 70 kg) minutes per bedposition. PET/MR scans were reconstructed using BSREM and factors *β* 150, 300, 450 and 600. List mode data with reduced counts were reconstructed to simulate scans with 17%, 33%, 50% and 67% activity reduction. Image quality was measured quantitatively for PET/CT and PET/MR phantom and patient data. Experienced nuclear medicine physicians performed visual image quality scoring and lesion counting in the PET/MR patient data.

**Results:**

Phantom analysis resulted in a possible injected activity reduction of 50% with factor *β* = 600. Quantitative analysis of patient images revealed a possible injected activity reduction of 67% with factor *β* = 600. Both with equal or improved image quality as compared to PET/CT. However, based on visual scoring a maximum activity reduction of 33% with factor *β* = 450 was acceptable, which was further limited by lesion detectability analysis to an injected activity reduction of 17% with factor *β* = 450.

**Conclusion:**

A digital [^68^Ga]Ga-DOTA-TATE PET/MR together with BSREM using factor *β* = 450 result in 17% injected activity reduction with quantitative values at least similar to analogue PET/CT, without compromising on PET/MR visual image quality and lesion detectability.

## Background

Neuroendocrine tumours (NETs) are a rare group of slow growing tumours, originating from neuroendocrine cells in predominantly the gastrointestinal tract and lungs. The majority of these tumours present a high number of somatostatin receptors (SSTR) on their cell surface. The presence of SSTR enables diagnosis and treatment with radiolabelled somatostatin analogues (SSA). At this moment, Positron Emission Tomography/Computed Tomography (PET/CT) using ^68^Gallium labeled somatostatin analogues ([^68^Ga]Ga-DOTA-SSA) plays a pivotal role in diagnosis and staging of patients with NETs [1, 2]. In the last decade, integrated PET/Magnetic Resonance (PET/MR) was introduced allowing for simultaneous PET and MR acquisitions. The most important advantage of PET/MR compared to PET/CT for patients with NETs is the higher soft tissue contrast of the MR part, especially in the liver, resulting in the detecting of more liver metastases [3–12]. Also, MR based attenuation correction (AC) does not increase radiation burden of the patient [13–16]. A drawback, however, is that MR-AC is less accurate than CT-AC and may result in a bias of the standard uptake value (SUV) [17–20]. Although, using time of flight (TOF) reconstructions improves the accuracy of PET/MR images [11, 20–25]. In this study an analogue Siemens Biograph mCT PET/CT (Siemens Healthineers AG, Erlangen, Germany) is compared with a digital GE Signa 3.0 T PET/MR (General Electric Healthcare, Milwaukee, WI, USA). The PET/MR system provides a higher sensitivity due to smaller detector ring diameter and the longer axial field of view (FOV) [26–29]. The simultaneous acquisition of PET and MR result in a higher temporal resolution [30, 31]. The digital silicon photomultiplier detectors (SiPM) of the PET/MR system enable faster TOF resolution [26, 29, 32–35]. Apart from these hardware factors, application of block-sequential regularized expectation maximization (BSREM) reconstruction algorithm on the PET/MR system, which uses a penalization factor *β* for noise suppression, improves contrast and lowers noise levels compared to ordered subset expectation maximum (OSEM3D) reconstructions [36–39]. These features can be exploited to achieve a reduction in amount of injected activity while maintaining diagnostic image quality. Several studies have investigated the possible reduction of the injected amount ^18^Fluorine-fluoro-2-deoxyglucose ([^18^F]FDG) activity for digital TOF PET/MR compared to analogue TOF PET/CT and found possible reductions of 37- 50% [25, 26, 29] using OSEM3D reconstructions. Currently it is unknown whether these findings with [^18^F]FDG may be directly extrapolated to [^68^Ga]Ga-DOTA-SSA PET/MR, because of differences in half-life, positron range, effect of magnetic field on positron range and biodistribution [40–43]. Therefore, the aim of this study is to determine the possible reduction in injected activity of [^68^Ga]Ga-DOTA-TATE for digital PET/MR with BSREM reconstruction while maintaining at least an equal diagnostic image quality compared to the current analogue PET/CT protocol.

## Methods

### Phantom preparation

A National Electrical Manufacturers Association (NEMA) image quality (IQ) phantom with sphere diameters 10, 13, 17, 22, 28 and 37 mm was scanned consecutively with sphere to background ratios of 10:1, 4:1, and 2:1 to assess the image quality. The spheres and background were filled with [^68^Ga]Ga-diethylene-triamine-pentaacetate ([^68^Ga]Ga-DTPA). The experiment started with a ratio of 10:1 and the spheres with 38.9 kBq/ml and the background with 4.0 kBq/ml at start of the PET/MR acquisition. After one and two hours extra activity was added to the background to achieve sphere to background ratios of 4:1 (22.1 kBq/ml: 5.2 kBq/ml) and 2:1 (12.0 kBq/ml: 5.4 kBq/ml) at the start of the PET/MR acquisition. Since PET/MR in patients is acquired with arms positioned downward adjacent to the body we also positioned two 500 ml plastic bottles filled with 0.32 kBq/ml beside the phantom during each PET/MR acquisition and an additional 10:1 PET/CT acquisition to simulate the attenuation and background signal from the arms.

### Phantom acquisition

Phantom acquisitions of two bed positions with acquisition times of 90 (10:1), 180 (4:1) and 360 (2:1) seconds were acquired with the clinical scan protocol on the GE Signa 3.0 T PET/MR (General Electric Healthcare, Milwaukee, WI, USA) and consecutively on the Siemens Biograph mCT PET/CT (Siemens Healthineers AG, Erlangen, Germany). Table 1 shows the most prominent system specifications of both PET cameras [40, 44–46]. Acquisition times for 4:1, 2:1 PET/MR and PET/CT were prolonged to compensate for radioactive decay, providing an equal amount of photon pairs for the spheres during all acquisitions.

**Table 1:** System specifications

	GE Signa PET/MR	Siemens Biograph mCT PET/CT
Scintilator	Lutetium‐yttrium oxyorthosilicate (LYSO)	Lutetium oxyorthosilicate (LSO)
Photodetector	SiPM	PMT
TOF (ps)	< 400	< 540
Axial view FOV (cm)	25	21.6
Trans-axial FOV(cm)	60	78
peak NECR (kcps @ kBq/ml)	216.8 @ 18.6	181.0 @ 25.2
Sensitivity at 10 cm offset (cps/kBq)	21	10

*PMT* photomultiplier, *ps* picoseconds, *FOV*: field of view, *NECR*: noise equivalent count rate, *cps*: counts per second, *kBq*: kilobecquerel

### Patient data

This study was approved by the Medical Ethical Committee of the Erasmus MC (MEC-2021–0209). Written informed consent was obtained from all the patients, and procedures were in accordance with the Declaration of Helsinki of 1964, as revised in 2013. Patients (n = 25) who were scheduled for ^68^Ga-DOTA-TATE PET/MR at the Erasmus MC for staging or restaging of NETs, were included; of whom five patients (n = 2 men, n = 3 women; mean age 38.2 ± 10.4 years) were included retrospectively between September 2020 and October 2021 and twenty (n = 8 men, n = 12 women: 48.6 ± 17.1 years) were included prospectively between February 2021 and February 2022. Inclusion criteria were: injected activity within 10% of the prescribed activity, PET acquisition time at 60 ± 6 min after injection. Patients with extensive liver tumour involvement were excluded. Subsequent scanning of the same patients on PET/CT and PET/MR was considered unethical by the COVID-19 regulations of our institution. To enable comparison of patient image quality between the scanners, previous (7 – 28 months prior) ^68^Ga-DOTA-TATE PET/CTs of six patients with stable disease (no additional lesions on PET/MR) were also included in the analysis.

### ^68^Ga-DOTA-TATE PET/MRI patient acquisition

Patients were prepared according to the same protocol used for the [^68^Ga]Ga-DOTA-TATE PET/CT. This protocol was optimized for sufficient image quality and lesion detectability in a previous study of Cox et al. [47]. Scans were planned just before the patients’ next scheduled monthly dose of long-acting somatostatin analogues (e.g., Sandostatine LAR, Novartis Pharma BV). Patients were stimulated to drink 1 l of water during the two hours before intravenous injection of 1.5 MBq/kg (mean 121.6 ± 24.5 MBq) [^68^Ga]Ga-DOTA-TATE [47]. PET/MR acquisitions were started 60 ± 3 min after tracer injection in supine position with the arms down. Whole body list mode PET images were acquired with an acquisition time of 3 (≤ 70 kg) or 4 (≥ 71 kg) minutes per bed position (min/bp). Simultaneously, standard MR sequences were acquired for attenuation correction (AC) (proton density weighted zero echo time (ZTE) and Dixon) and for anatomic correlation (T1-LAVA flex and T2-FrFSE Flex).

### ^68^Ga-DOTA-TATE PET/CT patient acquisition

PET/CT patient preparation, tracer injection and acquisition times were identical to the PET/MR exams. PET/CT scans were acquired in supine position with the arms positioned upward over the head according the protocol as used before by Cox et al. [47]. Acquisitions started at 60 ± 4 min after injection of 119.0 ± 22.0 MBq [^68^Ga]Ga-DOTA-TATE.

### Phantom image reconstruction

PET/MR data were reconstructed with BSREM (Q.Clear) using TOF, point spread function modelling (PSF) and all standard corrections, of which scatter and attenuation correction were applied with a modified attenuation map as the MR-AC algorithm is not suitable for phantom imaging [48]. This map was obtained using an in-house developed Python tool that combined a previously acquired CT based attenuation map of the phantom with manufacturer MR coil templates. Reconstructions were performed with noise penalization factors *β* of 150, 300, 450 and 600 with a matrix of 256 × 256 and a voxel size of 2.34 × 2.34 × 2.78 mm^3^. In addition, the list-mode data of the standard NEMA 4:1 scan were also reconstructed with reduced acquisition times to simulate PET scans with simulated activity reductions of 17%, 33%, 50%, and 67%.

PET/CT data were reconstructed using OSEM3D with PSF, TOF, all standard corrections, 3 iterations, 21 subsets, a 3 mm Gaussian post reconstruction filter, a matrix of 200 × 200 and a voxel size of 4.1 × 4.1 × 3.0 mm^3^.

### Patient image reconstruction

PET/MR data were corrected for attenuation and scatter with truncation completion and a hybrid ZTE/Dixon MR-AC method and reconstructed using the same parameters that were used in the phantom reconstructions. List-mode data were also reconstructed with reduced acquisition times to simulate injected activity reductions of 17%, 33%, 50%, and 67%. Patient PET/CT data were reconstructed as described for the phantom reconstructions.

### Phantom quantitative image quality analysis

The image quality measurements were based on the method proposed by Lindström et al. [36] and also used in other studies [39, 49]. As a measure of image noise, the background variability (BV) was calculated by dividing the standard deviation (SD) by the mean activity concentration in 6 cylindrical background volumes of interest (VOI) (diameter, ø = 27 mm × 70 mm). Spherical VOI’s matching the physical fillable volume were used for measurements of the spheres. The contrast-to-noise ratio (CNR) of each sphere was calculated by dividing contrast recovery (CR) (Eq. 1) by BV.

1 $${\text{CR}} = \frac{{{}_{{{\text{CB}}}}^{{\underline{{{\text{CH}}}} }} - {1}}}{{{}_{{{\text{aB}}}}^{{\underline{{{\text{aH}}}} }} - {1}}},$$

where CH and CB are counts in the spheres and background VOI’s and aH and aB, are the activities in the spheres and background VOI. Above image analysis was automated by an in-house developed Python script.

### Patient quantitative image quality analysis

Quantitative analysis of patient scans was performed using Hermes Hybrid viewer 2.6D software (Hermes Medical Solutions, Stockholm, Sweden). A VOI was placed in a lesion-free homogeneous part of the right liver lobe (diameter, ø = 3 cm) at least 1 cm from the edge of the liver to avoid partial volume effects. For each patient, VOIs were placed at the exact same location throughout all reconstructions and SD and liver mean standard uptake value (SUVmean) were measured. For up to three lesions per patient, a 50% threshold of SUVmax VOI or if this was not possible a fixed VOI within the lesion boundaries, was placed to measure lesion SUVmax. With these values, the following parameters were calculated according to method described by Lindström et al.[36] and adopted by other studies [39, 49, 50]:

2 $${\text{Liver}}\;{\text{noise}}\;{\text{normalized}}\;{\text{to}}\;{\text{liver}}\;{\text{SUVmean}}\;\left( {{\text{Liver}}\;{\text{noise}}} \right) = \frac{{{\text{Liver}}\;{\text{SD}}}}{{{\text{Liver}}\;{\text{SUVmean}}}},$$

3 $${\text{Lesion}}\;{\text{signal}}\;{\text{to}}\;{\text{noise}}\;{\text{ratio}}\;\left( {{\text{Lesion}}\;{\text{SNR}}} \right) = \frac{{{\text{Lesion}}\;{\text{SUVmax}}}}{{{\text{Liver}}\;{\text{noise}}}}{,}$$

4 $${\text{Lesion}}\;{\text{signal}}\;{\text{to}}\;{\text{background}}\;{\text{ratio}}\;\left( {{\text{Lesion}}\;{\text{SBR}}} \right){ = }\frac{{{\text{Lesion}}\;{\text{SUVmax}}}}{{{\text{Liver}}\;{\text{SUVmean}}}}{.}$$

### Visual image quality and lesion detectability analysis

The anonymized reconstructions were assessed by three readers. These experienced nuclear medicine physicians were blinded to patient data. Using OsiriX MD (Pixmeo SARL, Geneva, Switzerland) software the readers scored the reconstructions according to the method proposed by Halpern [51] and previously used in a similar setting [47]. The reconstructions were presented to the readers from 67 to 0% simulated activity reductions to avoid lesion recognition bias that might be introduced by viewing images with higher count statistics prior to images with lower count statistics. For subjective visual diagnostic image quality analysis, a four-point scoring scale was used: non-diagnostic (0), poor (1), moderate (2), or good (3). For lesion detectability, the physician recorded the number of somatostatin receptor (SSTR) positive lesions in all reconstructions for one selected body region (head/neck, thorax or abdomen) per patient. As reference, the number of lesions were compared with the 0% simulated activity reduction reconstruction with *β* = 300*.*

The visual analysis was performed in a two-step process, a method adapted from Bjöersdorff et al. [52] and Reynes et al. [53]. First, to find the optimal value for *β* for each simulated activity reduction step, lesion counting and scoring was performed by one reader for 20 reconstructions (4 different *β* values and 5 different levels of simulated activity reduction) of five random patients. For each simulated activity reduction step, images with a sufficient visual image quality (score ≥ 2) and lesion detectability (> 90%) were selected. If multiple *β* values per simulated activity reduction step were eligible, the reconstruction with the smallest difference in lesion SUVmax compared to the PET/CT was selected.

In the second step, lesion counting and scoring was conducted by two other readers for each simulated activity reduction and corresponding optimal *β*-value as obtained in the first step.

### Statistical analysis

Graphpad PRISM version 9 was used to test for significant differences in liver noise, lesion SNR, lesion SBR, lesion SUVmax between the clinical 0% simulated activity reduction with factor *β* = 300 or PET/CT reconstructions and the other simulated activity reduction reconstructions. A repeated measures analysis of variance (ANOVA) (*α* = 0.05) (including Mauchly’s test of sphericity with a Greenhouse–Geisser correction for nonsphericity) or a non-parametric Friedman test (*α* = 0.05) was performed, after testing the data for normality by a Shapiro–Wilk test (*α* = 0.05). Respectively, Dunnett’s or Dunn’s multiple comparison post hoc test was used to identify significant differences between reconstructions. These tests were also used to assess significant differences in visual scoring and lesion detectability of the final analysis between the 0% with factor *β* = 300 and other simulated activity reductions. IBM SPSS statistics version 28 was used for determining inter-rater reliability with Cohen’s kappa for visual scoring and with the intraclass correlation coefficient (ICC) for lesion detectability. ICC was calculated using a 2-way mixed-effects model with single rater and absolute-agreement [54].

## Results

### Phantom quantitative image quality analysis

**Fig. 1:**
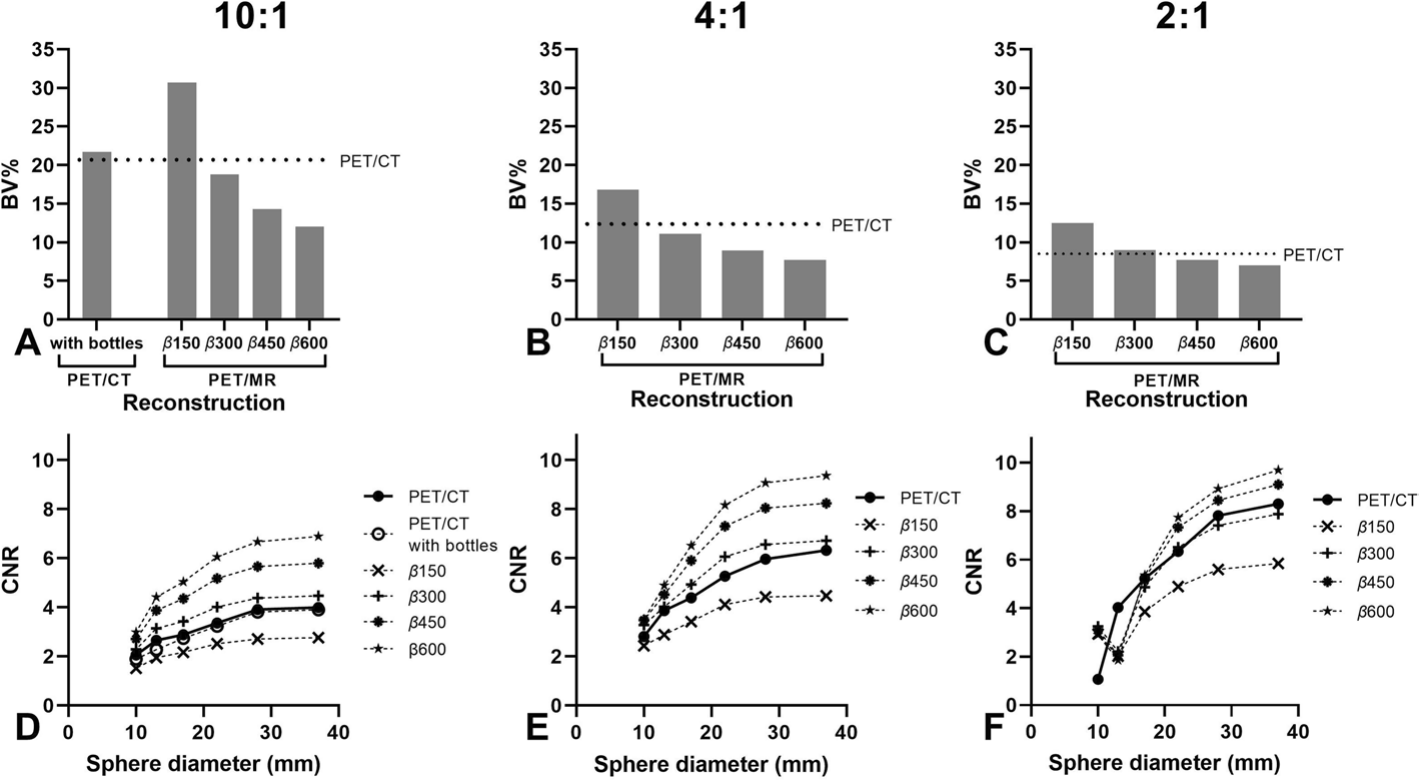
Phantom BV (**A**–**C**) and CNR (**D**–**F**) of the PET/CT OSEM3D reconstruction and the PET/MR BSREM 0% simulated activity reduction reconstructions with different values of factor *β.* Sphere to background ratios are 10:1 (**A** + **D**), 4:1 with (**B** + **E**) and 2:1 (**C** + **F**)

Figure 1 displays the results from the phantom study, showing BV and CNR measured in the PET/CT and the 0% simulated activity reduction PET/MR reconstructions for the different sphere to background ratios. Each increase of 150 in factor *β* (150 to 300, 300 to 450 and 450 to 600) results in a lower BV (on average about -35%, -21% and -14%) and a higher mean CNR (on average about + 35%, + 20% and + 14%) for all sphere to background ratios. PET/MR reconstructions with factor *β* ≥ 300 for ratio’s 4:1 and 10:1 outperform PET/CT for BV (≤ –9%) and mean CNR (≥ + 10%) and with factor *β* ≥ 450 for ratio 2:1 (≤ -16% (BV) and ≥  + 8% (mean CNR)), whereas reconstructions with factor *β* = 600 achieved the lowest BV and highest CNR. Furthermore, positioning of bottles next to the phantom results in a slightly higher BV (+ 5%) (Fig. 1A) and a slightly lower mean CNR (-5%) in PET/CT (Fig. 1D).

**Fig. 2:**
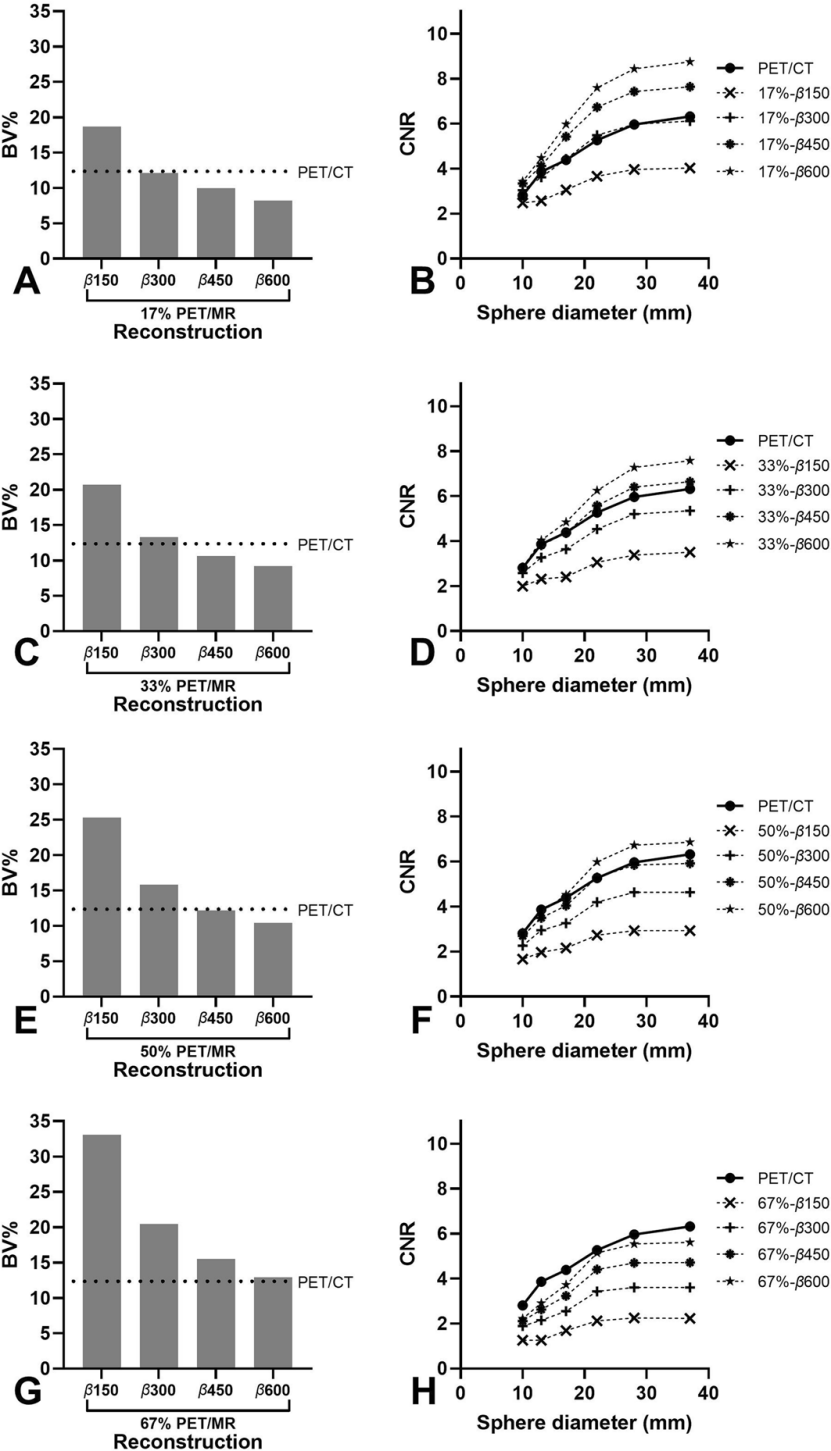
Comparing phantom BV (**A**, **C**, **E**, **G**) and CNR (**B**, **D**, **F**, **H**) of PET/CT OSEM3D reconstruction and PET/MR BSREM reconstructions with different values of factor *β* and simulated activity reductions of 17% (**A** + **B**), 33% (**C** + **D**), 50% (**E** + **F**) and 67% (**G** + **H**) in a sphere to background ratio of 4:1

The effect of simulated activity reduction on BV and CNR in PET/MR reconstructions with sphere to background ratio 4:1 is shown in Fig. 2. Lower BV and higher CNR than PET/CT can be seen in PET/MR reconstructions with factor *β* ≥ 300 for 17% (≤ -3% and ≥ 0%) simulated activity reduction (Fig. 2A + B), factor *β* ≥ 450 for 33% (≤ −15% and ≥  + 4%) simulated activity reduction (Fig. 2C + D). Whereas a simulated activity reduction of 50% (Fig. 2E + F) results in a lower BV (≤ −2%) with factor *β* ≥ 450, although CNR was lower (− 5%) for factor *β* = 450 and higher (+ 7%) for factor *β* = 600 compared to PET/CT. Figure 2E + H shows that a simulated activity reduction of 67% will result in higher BV (≥ + 3%) and lower CNR (≤ -53%) than PET/CT for all reconstructions.

### Patient quantitative image quality analysis

**Fig. 3:**
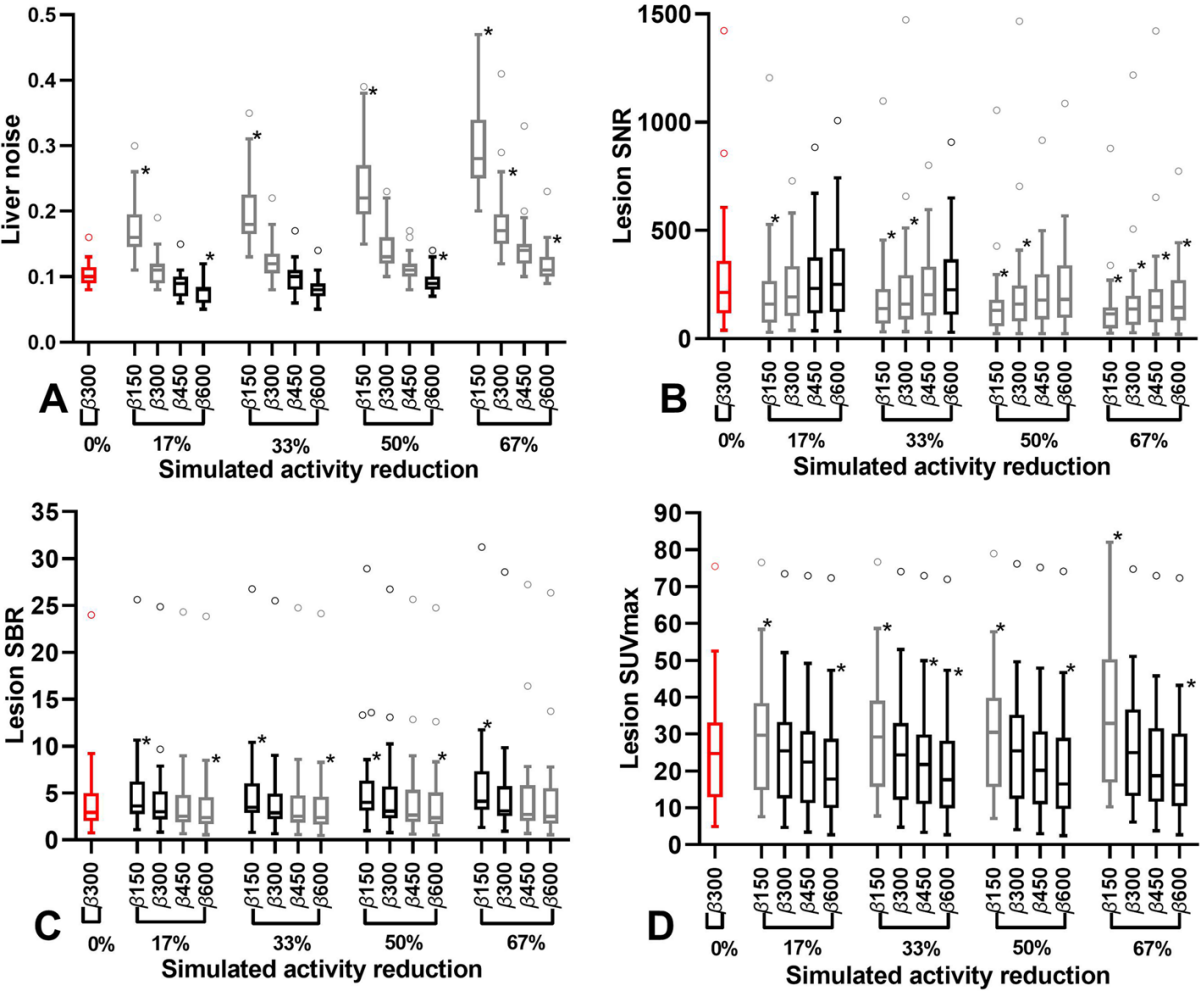
Box plots of liver noise (**A**), lesion SNR (**B**), lesion SBR (**C**) and lesion SUVmax comparison between 0% simulated activity reduction with factor *β* = 300 and 17%, 33%, 50% and 67% simulated activity reductions reconstructed with different values of factor *β* (150, 300, 450 and 600). Box plots of simulated activity reductions with equal or improved values compared to 0% simulated activity reduction with factor *β* = 300 (red) are displayed in black. The central line of the box plot represents the median value. The whiskers extent to the minimum and maximum values or 1.5 interquartile range (IQR) of the lower and the upper quartiles. Values outside the 1.5 IQR are plotted. Significant differences (*p* ≤ 0.05) compared to 0% simulated activity reduction with factor *β* = 300 determined by Friedman test with additional Dunn’s multiple comparisons test are indicated with asterisks

Figure 3A–D presents the comparison of quantitative results of 30 lesions (0.11–38 cm^3^) between the 0% simulated activity reduction with factor *β* = 300 and the other simulated activity reductions. As can be seen from the figure, each increase in factor *β* (150 to 300, 300 to 450 and 450 to 600) result in a decrease in liver noise (Fig. 3A on average about − 38%, − 22% and − 15%), lesion SBR (Fig. 3C on average about −15%, − 7% and − 6%) and lesion SUVmax (Fig. 3D on average about − 15%, − 9% and − 5%) for all simulated activity reductions, whereas lesion SNR (Fig. 3B on average about + 30%, + 14% and + 9%)) increased for all simulated activity reductions*.* Further analyze indicates that an increase of factor *β* to 450 (17% and 33%) and 600 (50%) is needed to maintain at least equal median liver noise (Fig. 3A). Although, to maintain at least equal median lesion SNR, a further increase of factor *β* is needed for ≥ 33% simulated activity reductions (Fig. 3B). In contrast to liver noise and lesion SNR, lesion SBR (Fig. 3C) and SUVmax (within 15%) (Fig. 3D) values remained similar with factor *β* = 300 for all simulated activity reductions.

**Fig. 4:**
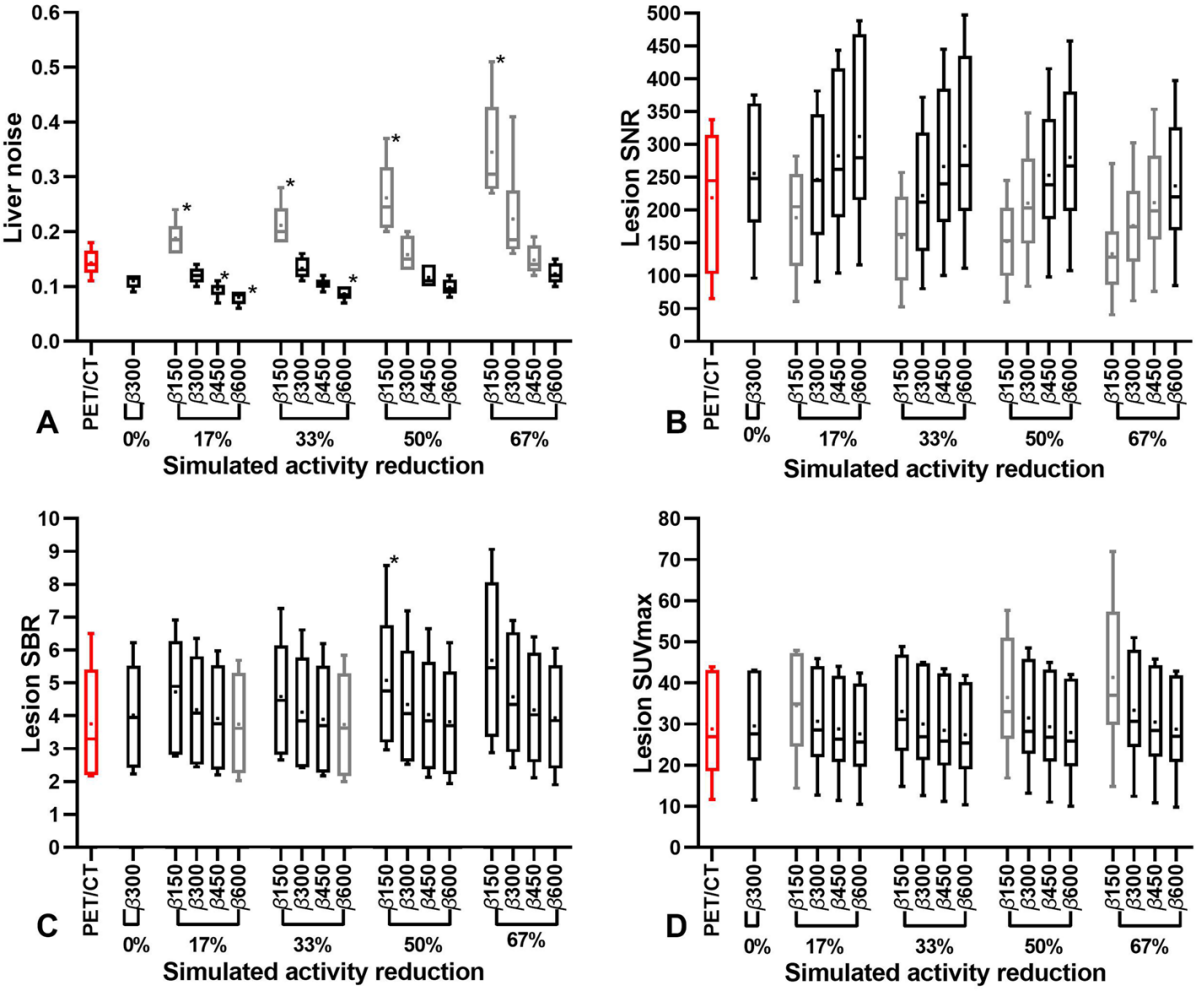
Box plots of liver noise (**A**), lesion SNR (**B**), lesion SBR (**C**) and lesion SUVmax comparison between PET/CT and simulated activity reductions (0%, 17%, 33%, 50% and 67%) with different values of factor *β* (150, 300, 450 and 600). Box plots of reconstructions with equal or improved values compared to PET/CT (red) are displayed in black. The central line of the box plot represents the median value and the square the mean value. The whiskers extent to the minimum and maximum values. Statistically significant differences (*p* ≤ 0.05) with PET/CT mean values determined by repeated measures ANOVA with additional Dunnett’s multiple comparisons test are indicated with asterisks

The quantitative results of the comparison between the PET/CT reconstruction and the simulated activity reductions of 6 patients with stable disease are displayed in Fig. 4. Figure 4A and B show that an increase of factor *β* = 300 is only required for 50% (*β* = 450) and 67% (*β* = 600) to obtain at least similar mean liver noise and mean lesion SNR than PET/CT. Whereas, lesion SBR (Fig. 4C) and lesion SUVmax (Fig. 4D) (within 15%) resulted in at least similar mean values compared to PET/CT with factor *β* = 300 for all simulated activity reductions.

### Visual image quality and lesion detectability analysis

**Fig. 5:**
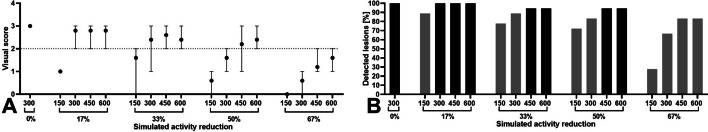
Visual score (**A**), simulated activity reductions with all scores ≥ 2 (sufficient image quality) can be found on or above the dashed line. Lesion detectability (**B**), simulated activity reductions with a lesion detectability ≥ 94% are displayed with black bars

Figure 5 shows the scoring and lesion counting for all reconstructions in five random patients. All lesions were detected and the visual image quality was scored as sufficient for 17% simulated activity reductions with factor *β* ≥ 300. For 33% with factor *β* ≥ 450 and 50% with factor *β* = 600 simulated activity reductions, sufficient image quality was obtained for while 94% of the lesions (one lesion not detected compared to reference number of lesions) were detected. Optimal *β*-values were determined at factor *β* = 450 for 17%, factor *β* = 450 for 33% and *β* = 600 for 50% simulated activity reductions. For 17% and 33% simulated activity reductions multiple factor *β-*values were eligible and optimal values were chosen based on SUVmax.

Table 2 provides an overview of the visual scores for each simulated activity reduction step with the optimal factor *β*-value for all patients per reader. As can be seen from the table, almost all simulated activity reductions scored at least moderate. The mean visual score was highest for 17% simulated activity reduction with factor *β* = 450, although this mean visual score was not significantly (*p* > 0.999) higher than 0% simulated activity reduction. Whereas, the mean visual score was significantly (*p* = 0.020) lower for 50% simulated activity with factor *β* = 600. Furthermore, the table shows fair inter-rater reliability between both readers for 17% simulated activity reduction with factor *β* = 450 and slight inter-rater reliability for 0% simulated activity reduction with factor *β* = 300.

**Table 2:** Visual image quality and interobserver inter-rater reliability for each simulated activity reduction

Simulated activity reduction	PET image quality									
	Nondiagnostic (0)		Poor (1)		Moderate (2)		Good (3)		Mean Score	Cohen’s Kappa
	Reader		Reader		Reader		Reader			
	1	2	1	2	1	2	1	2		
50%–β600	–	–	–	5	13	17	12	3	2.20*	0.05
33%–β450	–	–	–	1	19	9	6	15	2.40	0.04
17%–β450	–	–	–	1	10	2	15	22	2.72	0.27
0%–β300	–	–	–	–	17	3	8	22	2.60	0.12

Statistically significant differences (*p* ≤ 0.05) in mean visual score compared with 0% simulated activity reduction with factor *β* = 300 determined by Friedman test with additional Dunn’s multiple comparisons test are indicated with asterisks

The results of the lesion detectability analysis are displayed in Table 3. In contrast to visual scoring, lesion detectability analysis resulted in excellent inter-rater reliability between both readers. Similar to visual scoring, lesion detectability is significantly lower for 50% simulated activity reduction with factor *β* = 600 compared to 0% simulated activity reduction. Only 17% simulated activity reduction showed sufficient lesion detectability compared to 0% simulated activity reduction.

**Table 3:** Lesion detectability and inter-rater reliability for each simulated activity reduction

	Number of lesions			
	50%-β600*	33%-β450	17%-β450	0%-β300
Reader 1	68	67	74	76
Reader 2	58	68	73	77
ICC (95% CI)	0.96 (0.90–0.98)	0.98 (0.97–0.99)	0.97 (0.94–0.99)	0.97 (0.94–0.99)

Statistically significant differences (*p* ≤ 0.05) in mean visual score compared with 0% simulated activity reduction with factor *β* = 300 determined by Friedman test with additional Dunn’s multiple comparisons test are indicated with asterisks

## Discussion

The purpose of the present study was to determine the possible injected [^68^Ga]Ga-DOTA-TATE activity reduction for digital PET/MR with BSREM reconstruction while maintaining at least equal image quality compared to the current analogue PET/CT protocol. The results of this investigation show that digital PET/MR in combination with an increase of BSREM factor *β* up to 450 result in 17% injected activity reduction with quantitative values at least similar to analogue PET/CT, without compromising on PET/MR visual image quality and lesion detectability. Another major finding was the discrepancy of 50% versus 17% in possible activity reduction between the phantom and patient lesion detectability, which suggests that updating of the current NEMA IQ phantom is needed for subcentimeter lesion detection in digital PET imaging.

The NEMA IQ phantom study revealed a 50% (3–1.5 min/bp) [^68^Ga]Ga-DOTA-TATE BSREM PET/MR activity reduction by increasing factor *β* from 300 to 600 with at least comparable BV (− 17%) and CNR (+ 7%) values to analogue OSEM3D PET/CT. The finding that an increase in factor *β* enabled a reduction in injected activity was also observed in studies performed on a digital GE Discovery MI PET/CT scanner. This system is equipped with the same detectors, but in a 70 cm diameter bore configuration. Performance evaluation of a 5-ring (25 cm FOV) system resulted in comparable system specifications [55, 56] and overall image quality performance with GE Signa PET/MR [55]. Chicheportiche et al. [39] found with a 4-ring (20 cm FOV) system a possible activity reduction of 67% (1.5–0.5 min/bp) in combination with increase in factor *β* from 400 to 1000 resulted in equal or improved BV and CNR values compared to the GE OSEM3D reconstruction. Santoro et al. [57] proposed the CRBV function, that incorporates both the CR and BV as a measure of image quality and demonstrated with a 3-ring system (15 cm FOV) a possible activity reduction of 50% (4–2 min/bp) when increasing factor *β* from 300/350 to 500 (for spheres ≥ 17 mm) compared to the original OSEM3D reconstruction. [^68^Ga] phantom studies [49, 58–60] without activity reductions recommend optimal factor* β* values between 600 and 1000. In the current study, lower sphere to background ratios were found to cause an increase of partial volume effects (PVE) (Fig. 1F), which corroborates with previous studies of Krokos et al. [60] and a [^18^F] study of Yamaguchi et al. [61]. PVE affect mainly structures below 3 times full width half maximum (FWHM) of the reconstructed image resolution [62] (FWHM GE Signa PET/MR ± 4.2 mm [40]) as can be seen for 13 mm sphere in Fig. 1F.

Consistent with the phantom analysis, the quantitative analysis of 25 PET/MR patients revealed that an increase of factor *β* compensates higher liver noise and lower lesion SNR caused by injected activity reduction. However, increasing factor *β* reduced lesion SBR and lesion SUVmax. These findings are consistent with similar [^68^Ga] studies [39, 49, 63]. A comparison in 6 patients between OSEM3D (PSF + TOF 3i21s/3 mm) PET/CT and BSREM PET/MR showed a possible activity reduction of 67% using factor *β* = 600 without significant differences (*p* ≥ 0.05) in mean liver noise (-17%), lesion SNR (+ 8%), lesion SBR (+ 5%) and lesion SUVmax (0%). Subjective visual scoring analysis showed that the possible reduction was limited to 33% using *β* = 450 to ensure sufficient visual image quality. Although, the variation in visual scores between readers for each patient was large (Cohen’s Kappa = 0.04), in contrary to lesion detectability with very little variation (ICC = 0.97) in number of lesions between readers for each patient. This objective analysis further limits the injected activity reduction to 17% with factor *β* = 450 to ensure adequate lesion detectability (> 96% of lesions detected). Without significant differences (*p* ≥ 0.05) in lesion SNR (+ 29%), lesion SBR (+ 5%) and lesion SUVmax (0%) and a significant (*p* ≥ 0.04) improvement of mean liver noise (− 36%). These findings were again compared with two studies concerning both digital 4-ring Discovery MI PET/CT scanners with OSEM3D (PSF + TOF, 3i16s/5 mm) and BSREM reconstructions. A [^68^Ga]Ga-DOTA-TATE study of Chicheportiche et al. [39] (N = 8, 2 MBq/kg, 1.5 min/bp) found a possible reduction of 67% using factor *β* = 1200 with improved liver noise, lesion SBR and lesion SNR towards GE OSEM3D. This reduction was also found for overall visual image quality with *β* = 2400 (lowest value in visual analysis). Although, in accordance with the current study, this reduction was limited to 33% with factor *β* = 1400 to obtain sufficient lesion detectability. Consistent with the present study, a discrepancy of 33% in possible activity reduction between phantom and lesion detectability analysis was found. A possible explanation for this might be that both patient studies included subcentimeter lesions. In the current study, 50% of the lesions had an equal or lower volume than the 10 mm sphere (0.52 cm^3^). Therefore, their lesion detectability was not covered by the current NEMA IQ phantom analysis. This provides further support for updating the current NEMA IQ phantom for subcentimeter lesions as proposed by previous studies [59, 64–69]. A [^68^Ga]Ga-DOTA-TOC study (N = 13, 2.3 MBq/kg, 2 min/bp) of Lindström et al. [49] resulted in a comparable injected activity reduction of 25% with factor *β* = 533 with significantly (*p* < 0.001) improved liver noise, SNR and SBR, while SUVmax remained within 20% of OSEM3D values. In addition, they found possible activity reductions of 75% in ^68^Gallium prostate-specific membrane antigen ([^68^Ga]Ga-PSMA) (N = 20, 2 MBq/kg, 2 min/bp) with factor *β* = 1200 [63]. Other [^68^Ga] tracers studies [19, 58, 60, 70, 71] reported optimized factors *β* between 400 and 1600. This wide range is partly caused by differences in biodistribution of different tracers which results in other optimal factor *β* values as demonstrated above by Lindström et al. [49, 63] and also by and Baratto et al. [70]. This in combination with blurring of small lesions due the longer positron range of [^68^Ga] could also explain the need for different optimal factor *β* values for [^18^F]FDG [60]. Current study resulted in a lower factor *β* than other studies, which is related to several factors that result in a downward shift of this factor. The GE Signa has a higher count rate, sensitivity and spatial resolution than the PET/CT systems [40–43, 55, 72]. The quantitative comparison with an analogue PET/CT and OSEM3D reconstruction with higher number of subsets and lower Gaussian filter results in noisier reference PET/CT images. Furthermore, injected activity × acquisition time product of this study was higher than used by Chicheportiche et al. [39] resulting in a higher amount of signal and less noise. And lastly, the inclusion of a large amount of subcentimeter lesions, which obtain more improvement of lesion SNR. This PET/MR study was focused on injected activity reduction instead of acquisition time reduction, as the time needed for the simultaneous acquired MR sequences was at least 3 min/bp. In addition to this, prolonging PET acquisition times to match that of time-consuming MR sequences in dedicated regional PET/MR imaging enable further reduction in injected activity [73–75]. A weakness in this study was the use of a modified instead of a MR attenuation map for the phantom analysis, which might have affected the measurements and might contribute to the discrepancies between the phantom and patient analysis. Although, the discrepancy in possible injected activity reduction between phantom and patient analysis in current study was similar to the discrepancy found by the PET/CT study of Chicheportiche et al. [39]. The PET/CT patient comparison was limited to only a quantitative analysis of 6 older previous scans with sufficient image quality and equal number of lesions on PET/MR, instead of same day PET/CT scans with quantitative and visual analysis, as it was considered unethical to prolong hospital visits during the COVID-19 pandemic. The small sample and long period between the scans is a major limitation of this part of the study and makes the quantitative comparison results less reliable and generalizable. Although, the quantitative and visual comparison might also be affected by limitations of using DIXON based MR-AC for attenuation correction. Resulting in possible SUV bias in lesions near arms, metal implants, bone and air [17–19]. Nevertheless, several studies [11, 19–21, 24, 25] revealed that the TOF capability of the GE Signa compensates well for image quality loss and SUV bias due to attenuation. Although, further development and validation of more accurate MR-AC algorithms is an ongoing research topic. Unfortunately, the preferred SUVpeak analysis was not feasible due to the large amount of sub centimeter lesions. Instead of this, SUVmax analysis was used which is more prone to noise and PSF artifacts [52, 59, 76]. Since lesion detectability analysis was time consuming, a selection of only 4 reconstructions of 25 patients were serially analyzed from short to long acquisition times. This used method might introduce recognition bias but is commonly used in this kind of studies. This is the first [^68^Ga]Ga-DOTA-TATE GE Signa PET/MR with BSREM activity reduction study and results may not be valid for other scanners and reconstruction methods. Although, the evidence-based methods applied in this study can be used for future validation of other scanners, updated phantoms, optimization of guidelines and new reconstruction-based or deep learning approaches concerning PET image quality.

## Conclusions

A reduction of 17% [^68^Ga]Ga-DOTA-TATE injected activity in combination with BSREM factor *β* = 450 resulted in PET/MR images with quantitative values at least similar to the current PET/CT protocol, without compromising PET/MR visual image quality and lesion detectability.

## Data Availability

The data supporting our findings are available upon request.

## Abbreviations

BSREM: Block-sequential regularized expectation maximization
PET/MR: Positron emission tomography/magnetic resonance
PET/CT: Positron emission tomography/computed tomography
NETs: Neuroendocrine tumours
SSTR: Somatostatin receptor
SSA: Somatostatin analogues
[^68^Ga]Ga-DOTA-SSA: ^68^Gallium labeled somatostatin analogues
AC: Attenuation correction
FOV: Field of view
SiPM: Silicon photomultiplier detectors
TOF: Time-of-flight
OSEM3D: Ordered subset expectation maximum
[18F]FDG: ^18^Fluorine-fluoro-2-deoxyglucose
[^68^Ga]Ga-DOTA-TATE: ^68^Gallium-DOTATyr3-Octreotate
NEMA: National Electrical Manufacturers Association
IQ: Image quality
[^68^Ga]Ga-DTPA: [^68^Ga]Ga-diethylene-triamine-pentaacetate
kBq/ml: Kilobequerel/millilitre
LBS: Lutetium-based scintillator
LSO: Lutetium oxyorthosilicate
PMT: Photomultiplier
ps: Picoseconds
NECR: Noise equivalent count rate
cps: Counts per second
Min/bp: Minutes per bed position
ZTE: Zero echo time
PSF: Point spread function
BV: Background variability
SD: Standard deviation
VOI: Volumes of interest
CNR: Contrast-to-noise ratio
CR: Contrast recovery
SUV: Standard uptake value
Lesion SNR: Lesion signal to noise ratio
Lesion SBR: Lesion signal to background ratio
ANOVA: Analysis of variance
ICC: Intraclass correlation coefficient
PVE: Partial volume effects
FWHM: Full width half maximum
[^68^Ga]Ga-PSMA: ^68^Gallium prostate-specific membrane antigen

## References

[1] Deppen SA, Liu E, Blume JD, Clanton J, Shi C, Jones-Jackson LB, et al. Safety and efficacy of 68Ga-DOTATATE PET/CT for diagnosis, staging, and treatment management of neuroendocrine tumors. J Nucl Med. 2016;57(5):708–14. 26769865 10.2967/jnumed.115.163865 PMC5362940

[2] Hofman MS, Kong G, Neels OC, Eu P, Hong E, Hicks RJ. High management impact of Ga-68 DOTATATE (GaTate) PET/CT for imaging neuroendocrine and other somatostatin expressing tumours. J Med Imaging Radiat Oncol. 2012;56(1):40–7. 22339744 10.1111/j.1754-9485.2011.02327.x

[3] Mayerhoefer ME, Prosch H, Beer L, Tamandl D, Beyer T, Hoeller C, et al. PET/MRI versus PET/CT in oncology: a prospective single-center study of 330 examinations focusing on implications for patient management and cost considerations. Eur J Nucl Med Mol Imaging. 2020;47(1):51–60. 31410538 10.1007/s00259-019-04452-y PMC6885019

[4] Alshammari A. Impact of integrated whole body 68Ga PET/MR imaging in comparison with 68Ga PET/CT in Lesions detection and diagnosis of suspected neuroendocrine tumours. Am J Intern Med. 2019;7(4):102–11.

[5] Sawicki LM, Deuschl C, Beiderwellen K, Ruhlmann V, Poeppel TD, Heusch P, et al. Evaluation of (68)Ga-DOTATOC PET/MRI for whole-body staging of neuroendocrine tumours in comparison with (68)Ga-DOTATOC PET/CT. Eur Radiol. 2017;27(10):4091–9. 28439648 10.1007/s00330-017-4803-2

[6] Jawlakh H, Velikyan I, Welin S, Sundin A. (68) Ga-DOTATOC-PET/MRI and (11) C-5-HTP-PET/MRI are superior to (68) Ga-DOTATOC-PET/CT for neuroendocrine tumour imaging. J Neuroendocrinol. 2021;33(6):e12981. 34046974 10.1111/jne.12981

[7] Rajamohan N, Khasawneh H, Singh A, Suman G, Johnson GB, Majumder S, et al. PET/CT and PET/MRI in neuroendocrine neoplasms. Abdom Radiol (NY). 2022;47(12):4058–72. 35426497 10.1007/s00261-022-03516-2 PMC12994855

[8] Hope TA, Pampaloni MH, Nakakura E, VanBrocklin H, Slater J, Jivan S, et al. Simultaneous (68)Ga-DOTA-TOC PET/MRI with gadoxetate disodium in patients with neuroendocrine tumor. Abdom Imaging. 2015;40(6):1432–40. 25820755 10.1007/s00261-015-0409-9

[9] Beiderwellen KJ, Poeppel TD, Hartung-Knemeyer V, Buchbender C, Kuehl H, Bockisch A, et al. Simultaneous 68Ga-DOTATOC PET/MRI in patients with gastroenteropancreatic neuroendocrine tumors: initial results. Invest Radiol. 2013;48(5):273–9. 23493121 10.1097/RLI.0b013e3182871a7f

[10] Buchbender C, Heusner TA, Lauenstein TC, Bockisch A, Antoch G. Oncologic PET/MRI, part 1: tumors of the brain, head and neck, chest, abdomen, and pelvis. J Nucl Med. 2012;53(6):928–38. 22582048 10.2967/jnumed.112.105338

[11] Minamimoto R, Iagaru A, Jamali M, Holley D, Barkhodari A, Vasanawala S, et al. Conspicuity of malignant Lesions on PET/CT and simultaneous time-of-flight PET/MRI. PLoS ONE. 2017;12(1):e0167262. 28103230 10.1371/journal.pone.0167262 PMC5245859

[12] Reiner CS, Stolzmann P, Husmann L, Burger IA, Hüllner MW, Schaefer NG, et al. Protocol requirements and diagnostic value of PET/MR imaging for liver metastasis detection. Eur J Nucl Med Mol Imaging. 2014;41(4):649–58. 24346415 10.1007/s00259-013-2654-x

[13] Catana C. Principles of simultaneous PET/MR imaging. Magn Reson Imaging Clin N Am. 2017;25(2):231–43. 28390525 10.1016/j.mric.2017.01.002 PMC5385858

[14] Schafer JF, Gatidis S, Schmidt H, Guckel B, Bezrukov I, Pfannenberg CA, et al. Simultaneous whole-body PET/MR imaging in comparison to PET/CT in pediatric oncology: initial results. Radiology. 2014;273(1):220–31. 24877983 10.1148/radiol.14131732

[15] Gatidis S, Schmidt H, Gucke B, Bezrukov I, Seitz G, Ebinger M, et al. Comprehensive oncologic imaging in infants and preschool children with substantially reduced radiation exposure using combined simultaneous (1)(8)F-Fluorodeoxyglucose positron emission tomography/magnetic resonance imaging: a direct comparison to (1)(8)F-fluorodeoxyglucose positron emission tomography/computed tomography. Invest Radiol. 2016;51(1):7–14. 26309185 10.1097/RLI.0000000000000200

[16] Martin O, Schaarschmidt BM, Kirchner J, Suntharalingam S, Grueneisen J, Demircioglu A, et al. PET/MRI versus PET/CT for whole-body staging: results from a single-center observational study on 1,003 sequential examinations. J Nucl Med. 2020;61(8):1131–6. 31806777 10.2967/jnumed.119.233940

[17] Krokos G, MacKewn J, Dunn J, Marsden P. A review of PET attenuation correction methods for PET-MR. EJNMMI Physics. 2023;10(1):52. 37695384 10.1186/s40658-023-00569-0 PMC10495310

[18] Catana C. Attenuation correction for human PET/MRI studies. Phys Med Biol. 2020;65(23):23TR02. 33263313 10.1088/1361-6560/abb0f8 PMC8590198

[19] Ter Voert E, Muehlematter UJ, Delso G, Pizzuto DA, Müller J, Nagel HW, et al. Quantitative performance and optimal regularization parameter in block sequential regularized expectation maximization reconstructions in clinical (68)Ga-PSMA PET/MR. EJNMMI Res. 2018;8(1):70. 30054750 10.1186/s13550-018-0414-4 PMC6063806

[20] Tanaka A, Sekine T, Ter Voert E, Zeimpekis KG, Delso G, de Galiza BF, et al. Reproducibility of Standardized Uptake Values Including Volume Metrics Between TOF-PET-MR and TOF-PET-CT. Front Med (Lausanne). 2022;9:796085. 35308500 10.3389/fmed.2022.796085 PMC8924656

[21] Davison H, ter Voert EE, de Galiza BF, Veit-Haibach P, Delso G. Incorporation of Time-of-Flight Information Reduces Metal Artifacts in Simultaneous Positron Emission Tomography/Magnetic Resonance Imaging: A Simulation Study. Invest Radiol. 2015;50(7):423–9. 25756682 10.1097/RLI.0000000000000146

[22] Mehranian A, Zaidi H. Impact of time-of-flight PET on quantification errors in MR imaging-based attenuation correction. J Nucl Med. 2015;56(4):635–41. 25745090 10.2967/jnumed.114.148817

[23] Svirydenka H, Delso G, De Galiza BF, Huellner M, Davison H, Fanti S, et al. The Effect of Susceptibility Artifacts Related to Metallic Implants on Adjacent-Lesion Assessment in Simultaneous TOF PET/MR. J Nucl Med. 2017;58(7):1167–73. 28062597 10.2967/jnumed.116.180802

[24] Vontobel J, Liga R, Possner M, Clerc OF, Mikulicic F, Veit-Haibach P, et al. MR-based attenuation correction for cardiac FDG PET on a hybrid PET/MRI scanner: comparison with standard CT attenuation correction. Eur J Nucl Med Mol Imaging. 2015;42(10):1574–80. 26091704 10.1007/s00259-015-3089-3

[25] Zeimpekis KG, Barbosa F, Hullner M, ter Voert E, Davison H, Veit-Haibach P, et al. Clinical evaluation of PET image quality as a function of acquisition time in a new TOF-PET/MRI compared to TOF-PET/CT–initial results. Mol Imaging Biol. 2015;17(5):735–44. 25840683 10.1007/s11307-015-0845-5

[26] Queiroz MA, Delso G, Wollenweber S, Deller T, Zeimpekis K, Huellner M, et al. Dose optimization in TOF-PET/MR compared to TOF-PET/CT. PLoS ONE. 2015;10(7):e0128842. 26147919 10.1371/journal.pone.0128842 PMC4493146

[27] Karlberg AM, Sæther O, Eikenes L, Goa PE. Quantitative comparison of PET performance-Siemens biograph mCT and mMR. EJNMMI Phys. 2016;3(1):5. 26911722 10.1186/s40658-016-0142-7 PMC4766138

[28] Delso G, Martinez-Möller A, Bundschuh RA, Nekolla SG, Ziegler SI. The effect of limited MR field of view in MR/PET attenuation correction. Med Phys. 2010;37(6):2804–12. 20632591 10.1118/1.3431576

[29] Sekine T, Delso G, Zeimpekis KG, de Galiza BF, Ter Voert E, Huellner M, et al. Reduction of (18)F-FDG dose in clinical PET/MR imaging by using silicon photomultiplier detectors. Radiology. 2018;286(1):249–59. 28914600 10.1148/radiol.2017162305

[30] Al-Nabhani KZ, Syed R, Michopoulou S, Alkalbani J, Afaq A, Panagiotidis E, O’Meara C, et al. Qualitative and quantitative comparison of PET/CT and PET/MR imaging in clinical practice PET/MRI: Technical Challenges and Recent Advances Improving the detection of small lesions using a state-of-the-art time-of-flight PET/CT system and small-voxel reconstructions. J Nucl Med. 2014;55(1):88–94. 24337608 10.2967/jnumed.113.123547

[31] Alexander D, Michael S, Matthias E, Ambros JB, Sebastian F, Axel M-M, et al. First clinical experience with integrated whole-body PET/MR: comparison to PET/CT in patients with oncologic diagnoses. J Nucl Med. 2012;53(6):845–55. 22534830 10.2967/jnumed.111.098608

[32] Behr SC, Bahroos E, Hawkins RA, Nardo L, Ravanfar V, Capbarat EV, et al. Quantitative and visual assessments toward potential sub-mSv or ultrafast FDG PET using high-sensitivity TOF PET in PET/MRI. Mol Imaging Biol. 2018;20(3):492–500. 29192363 10.1007/s11307-017-1145-z

[33] Levin CS, Maramraju SH, Khalighi MM, Deller TW, Delso G, Jansen F. Design features and mutual compatibility studies of the time-of-flight PET capable GE SIGNA PET/MR system. IEEE Trans Med Imaging. 2016;35(8):1907–14. 26978664 10.1109/TMI.2016.2537811

[34] Zhang J, Maniawski P, Knopp MV. Performance evaluation of the next generation solid-state digital photon counting PET/CT system. EJNMMI Res. 2018;8(1):97. 30402779 10.1186/s13550-018-0448-7 PMC6219999

[35] Chicheportiche A, Marciano R, Orevi M. Comparison of NEMA characterizations for discovery MI and discovery MI-DR TOF PET/CT systems at different sites and with other commercial PET/CT systems. EJNMMI Phys. 2020;7(1):4. 31938953 10.1186/s40658-020-0271-x PMC6960280

[36] Lindstrom E, Sundin A, Trampal C, Lindsjo L, Ilan E, Danfors T, et al. Evaluation of penalized-likelihood estimation reconstruction on a digital time-of-flight PET/CT Scanner for (18)F-FDG whole-body examinations. J Nucl Med. 2018;59(7):1152–8. 29449445 10.2967/jnumed.117.200790

[37] Parvizi N, Franklin JM, McGowan DR, Teoh EJ, Bradley KM, Gleeson FV. Does a novel penalized likelihood reconstruction of 18F-FDG PET-CT improve signal-to-background in colorectal liver metastases? Eur J Radiol. 2015;84(10):1873–8. 26163992 10.1016/j.ejrad.2015.06.025

[38] Tragardh E, Minarik D, Almquist H, Bitzen U, Garpered S, Hvittfelt E, et al. Impact of acquisition time and penalizing factor in a block-sequential regularized expectation maximization reconstruction algorithm on a Si-photomultiplier-based PET-CT system for (18)F-FDG. EJNMMI Res. 2019;9(1):64. 31342214 10.1186/s13550-019-0535-4 PMC6656834

[39] Chicheportiche A, Goshen E, Godefroy J, Grozinsky-Glasberg S, Oleinikov K, Meirovitz A, et al. Can a penalized-likelihood estimation algorithm be used to reduce the injected dose or the acquisition time in (68)Ga-DOTATATE PET/CT studies? EJNMMI Phys. 2021;8(1):13. 33580359 10.1186/s40658-021-00359-6 PMC7881076

[40] Caribé P, Koole M, D’Asseler Y, Deller TW, Van Laere K, Vandenberghe S. NEMA NU 2–2007 performance characteristics of GE Signa integrated PET/MR for different PET isotopes. EJNMMI Phys. 2019;6(1):11. 31273558 10.1186/s40658-019-0247-x PMC6609673

[41] Caribé P, Vandenberghe S, Diogo A, Pérez-Benito D, Efthimiou N, Thyssen C, et al. Monte Carlo simulations of the GE Signa PET/MR for different radioisotopes. Front Physiol. 2020;11:525575. 33041852 10.3389/fphys.2020.525575 PMC7522581

[42] Grant AM, Deller TW, Khalighi MM, Maramraju SH, Delso G, Levin CS. NEMA NU 2–2012 performance studies for the SiPM-based ToF-PET component of the GE SIGNA PET/MR system. Med Phys. 2016;43(5):2334. 27147345 10.1118/1.4945416

[43] Huang SY, Savic D, Yang J, Shrestha U, Seo Y. The effect of magnetic field on positron range and spatial resolution in an integrated whole-body time-of-flight PET/MRI system. IEEE Nucl Sci Symp Conf Rec (1997); 2014. 10.1109/NSSMIC.2014.7431006 PMC4828037 27076778

[44] Healthcare. G. SIGNA™ PET/MR Technical Data.

[45] Healthineers S. Biograph mCT Technical Specifications.

[46] Reddin JS, Scheuermann JS, Bharkhada D, Smith AM, Casey ME, Conti M, et al., editors. Performance Evaluation of the SiPM-based Siemens Biograph Vision PET/CT System. In: 2018 IEEE Nuclear Science Symposium and Medical Imaging Conference Proceedings (NSS/MIC); 10–17 Nov. 2018.

[47] Cox CPW, Segbers M, Graven LH, Brabander T, van Assema DME. Standardized image quality for (68)Ga-DOTA-TATE PET/CT. EJNMMI Res. 2020;10:27. 32201912 10.1186/s13550-020-0601-y PMC7085989

[48] Boellaard R, Rausch I, Beyer T, Delso G, Yaqub M, Quick HH, et al. Quality control for quantitative multicenter whole-body PET/MR studies: a NEMA image quality phantom study with three current PET/MR systems. Med Phys. 2015;42(10):5961–9. 26429271 10.1118/1.4930962

[49] Lindström E, Lindsjö L, Sundin A, Sörensen J, Lubberink M. Evaluation of block-sequential regularized expectation maximization reconstruction of (68)Ga-DOTATOC, (18)F-fluoride, and (11)C-acetate whole-body examinations acquired on a digital time-of-flight PET/CT scanner. EJNMMI Phys. 2020;7(1):40. 32542512 10.1186/s40658-020-00310-1 PMC7295929

[50] Dwivedi P, Sawant V, Vajarkar V, Vatsa R, Choudhury S, Jha AK, et al. Analysis of image quality by regulating beta function of BSREM reconstruction algorithm and comparison with conventional reconstructions in carcinoma breast studies of PET CT with BGO detector. Nucl Med Commun. 2023;44(1):56–64. 36449665 10.1097/MNM.0000000000001631

[51] Halpern BS, Dahlbom M, Auerbach MA, Schiepers C, Fueger BJ, Weber WA, et al. Optimizing imaging protocols for overweight and obese patients: a lutetium orthosilicate PET/CT study. J Nucl Med. 2005;46(4):603–7. 15809482

[52] Bjöersdorff M, Oddstig J, Karindotter-Borgendahl N, Almquist H, Zackrisson S, Minarik D, et al. Impact of penalizing factor in a block-sequential regularized expectation maximization reconstruction algorithm for (18)F-fluorocholine PET-CT regarding image quality and interpretation. EJNMMI Phys. 2019;6(1):5. 30900064 10.1186/s40658-019-0242-2 PMC6428870

[53] Reynés-Llompart G, Gámez-Cenzano C, Vercher-Conejero JL, Sabaté-Llobera A, Calvo-Malvar N, Martí-Climent JM. Phantom, clinical, and texture indices evaluation and optimization of a penalized-likelihood image reconstruction method (Q.Clear) on a BGO PET/CT scanner. Med Phys. 2018;45(7):3214–22. 29782657 10.1002/mp.12986

[54] Koo TK, Li MY. A guideline of selecting and reporting intraclass correlation coefficients for reliability research. J Chiropr Med. 2016;15(2):155–63. 27330520 10.1016/j.jcm.2016.02.012 PMC4913118

[55] Pan T, Einstein SA, Kappadath SC, Grogg KS, Lois Gomez C, Alessio AM, et al. Performance evaluation of the 5-Ring GE Discovery MI PET/CT system using the national electrical manufacturers association NU 2–2012 Standard. Med Phys. 2019;46(7):3025–33. 31069816 10.1002/mp.13576 PMC7251507

[56] Zeimpekis KG, Kotasidis FA, Huellner M, Nemirovsky A, Kaufmann PA, Treyer V. NEMA NU 2–2018 performance evaluation of a new generation 30-cm axial field-of-view Discovery MI PET/CT. Eur J Nucl Med Mol Imaging. 2022;49(9):3023–32. 35284970 10.1007/s00259-022-05751-7 PMC9250480

[57] Santoro M, Della Gala G, Paolani G, Zagni F, Civollani S, Strolin S, et al. A novel figure of merit to investigate (68)Ga PET/CT image quality based on patient weight and lesion size using QClear reconstruction algorithm: a phantom study. Phys Med. 2023;106:102523. 36641902 10.1016/j.ejmp.2022.102523

[58] Seo Y, Khalighi MM, Wangerin KA, Deller TW, Wang YH, Jivan S, et al. Quantitative and qualitative improvement of low-count [(68)Ga]Citrate and [(90)Y]Microspheres PET image reconstructions using block sequential regularized expectation maximization algorithm. Mol Imaging Biol. 2020;22(1):208–16. 30993558 10.1007/s11307-019-01347-0 PMC6800603

[59] Rijnsdorp S, Roef MJ, Arends AJ. Impact of the noise penalty factor on quantification in bayesian penalized likelihood (Q.Clear) reconstructions of (68)Ga-PSMA PET/CT Scans. Diagnostics. 2021;11(5):847. 34066854 10.3390/diagnostics11050847 PMC8150604

[60] Krokos G, Pike LC, Cook GJR, Marsden PK. Standardisation of conventional and advanced iterative reconstruction methods for Gallium-68 multi-centre PET-CT trials. EJNMMI Phys. 2021;8(1):52. 34273020 10.1186/s40658-021-00400-8 PMC8286213

[61] Yamaguchi S, Wagatsuma K, Miwa K, Ishii K, Inoue K, Fukushi M. Bayesian penalized-likelihood reconstruction algorithm suppresses edge artifacts in PET reconstruction based on point-spread-function. Phys Med. 2018;47:73–9. 29609821 10.1016/j.ejmp.2018.02.013

[62] Marine S, Stephen LB, Irène B. Partial-Volume Effect in PET Tumor Imaging. J Nucl Med. 2007;48(6):932–45. 17504879 10.2967/jnumed.106.035774

[63] Lindström E, Velikyan I, Regula N, Alhuseinalkhudhur A, Sundin A, Sörensen J, et al. Regularized reconstruction of digital time-of-flight (68)Ga-PSMA-11 PET/CT for the detection of recurrent disease in prostate cancer patients. Theranostics. 2019;9(12):3476–84. 31281491 10.7150/thno.31970 PMC6587171

[64] Øen SK, Aasheim LB, Eikenes L, Karlberg AM. Image quality and detectability in Siemens Biograph PET/MRI and PET/CT systems-a phantom study. EJNMMI Phys. 2019;6(1):16. 31385052 10.1186/s40658-019-0251-1 PMC6682841

[65] Lu S, Zhang P, Li C, Sun J, Liu W, Zhang P. A NIM PET/CT phantom for evaluating the PET image quality of micro-lesions and the performance parameters of CT. BMC Med Imaging. 2021;21(1):165. 34749660 10.1186/s12880-021-00683-4 PMC8576981

[66] Tian D, Yang H, Li Y, Cui B, Lu J. The effect of Q.Clear reconstruction on quantification and spatial resolution of 18F-FDG PET in simultaneous PET/MR. EJNMMI Phys. 2022;9(1):1. 35006411 10.1186/s40658-021-00428-w PMC8748582

[67] Hashimoto N, Morita K, Tsutsui Y, Himuro K, Baba S, Sasaki M. Time-of-flight information improved the detectability of subcentimeter spheres using a clinical PET/CT scanner. J Nucl Med Technol. 2018;46(3):268–73. 29599404 10.2967/jnmt.117.204735

[68] Adler S, Seidel J, Choyke P, Knopp MV, Binzel K, Zhang J, et al. Minimum lesion detectability as a measure of PET system performance. EJNMMI Phys. 2017;4(1):13. 28260215 10.1186/s40658-017-0179-2 PMC5337231

[69] Miwa K, Wagatsuma K, Nemoto R, Masubuchi M, Kamitaka Y, Yamao T, et al. Detection of sub-centimeter lesions using digital TOF-PET/CT system combined with Bayesian penalized likelihood reconstruction algorithm. Ann Nucl Med. 2020;34(10):762–71. 32623569 10.1007/s12149-020-01500-8

[70] Baratto L, Duan H, Ferri V, Khalighi M, Iagaru A. The effect of various β values on image quality and semiquantitative measurements in 68Ga-RM2 and 68Ga-PSMA-11 PET/MRI images reconstructed with a block sequential regularized expectation maximization algorithm. Clin Nucl Med. 2020;45(7):506–13. 32433170 10.1097/RLU.0000000000003075

[71] Zanoni L, Argalia G, Fortunati E, Malizia C, Allegri V, Calabrò D, et al. Can QClear reconstruction be used to improve [68Ga]Ga-DOTANOC PET/CT image quality in overweight NEN patients? Eur J Nucl Med Mol Imaging. 2022;49(5):1607–12. 34693467 10.1007/s00259-021-05592-w

[72] David FCH, Ezgi I, William TP, Jorge U, Mark L, Craig SL. Studies of a next-generation silicon-photomultiplier–based time-of-flight PET/CT system. J Nucl Med. 2017;58(9):1511. 28450566 10.2967/jnumed.117.189514

[73] Svirydenka H, Muehlematter UJ, Nagel HW, Delso G, Ferraro DA, Kudura K, et al. 68Ga-PSMA-11 dose reduction for dedicated pelvic imaging with simultaneous PET/MR using TOF BSREM reconstructions. Eur Radiol. 2020;30(6):3188–97. 32060711 10.1007/s00330-020-06667-2

[74] Soret M, Maisonobe J-A, Desarnaud S, Bergeret S, Causse-Lemercier V, Berenbaum A, et al. Ultra-low-dose in brain 18F-FDG PET/MRI in clinical settings. Sci Rep. 2022;12(1):15341. 36097015 10.1038/s41598-022-18029-7 PMC9467977

[75] Oehmigen M, Ziegler S, Jakoby BW, Georgi JC, Paulus DH, Quick HH. Radiotracer dose reduction in integrated PET/MR: implications from national electrical manufacturers association phantom studies. J Nucl Med. 2014;55(8):1361–7. 25006216 10.2967/jnumed.114.139147

[76] Munk OL, Tolbod LP, Hansen SB, Bogsrud TV. Point-spread function reconstructed PET images of sub-centimeter lesions are not quantitative. EJNMMI Phys. 2017;4(5):1–2. 28091957 10.1186/s40658-016-0169-9 PMC5236043

